# Wither the self-sufficiency illusion? Food security in Arab Gulf States and the impact of COVID-19

**DOI:** 10.1007/s12571-020-01081-4

**Published:** 2020-07-08

**Authors:** Eckart Woertz

**Affiliations:** German Institute for Global and Area Studies (GIGA) and University of Hamburg, Hamburg, Germany

**Keywords:** Middle East and North Africa, Food security, Land investments, Value chains, Safety nets

## Abstract

Past approaches to food security in the countries of the Gulf Cooperation Council (GCC) were informed by concerns about food availability. They aimed at domestic self-sufficiency and self-sufficiency by proxy (via farmland investments abroad). These strategies have failed. Water scarcity at home increasingly compromises agricultural production. Farmland investments abroad have not matched ambitious related announcements due to a complex mixture of commercial, socio-economic and political factors. They do not contribute meaningful quantities to the Gulf countries’ food imports. The failure of such strategies has prompted a shift of focus instead towards value chain management as a means to secure food availability. Rather than trying to fight food import dependence, the Gulf countries now accept and manage it. However, malnutrition that leads to high levels of obesity and diabetes constitutes a risk factor in the face of COVID-19. Food accessibility for vulnerable population segments such as migrant labour is another issue that requires yet further policy measures, such as safety nets – whose expansion would be politically controversial if not impossible, however.

## Introduction

The Middle East and North Africa (MENA) region is the largest importer of cereals globally. Its imports of poultry and dairy products are extensive as well. Only with regard to fruit and vegetables do some countries of the region have a meaningful degree of self-sufficiency, or are even net exporters – such as Turkey and Morocco. The vulnerability to import disruptions is most acutely felt in the arid Gulf region that is rich in oil but poor in water and agricultural potential.

To alleviate such fears, Saudi Arabia and the United Arab Emirates (UAE) expanded local food production with the help of unsustainable mining of fossil water after the world food crisis of 1972–1975. By the time of the global food crisis of 2007/8 this strategy had run its course, as aquifers ran dry. This time, the Gulf countries rushed to secure privileged bilateral access to food production abroad. Large farmland investments were announced, often in food-insecure countries such as Ethiopia and Sudan.

The COVID-19 crisis and its impact on global food systems is an opportunity to revisit these past approaches to food security. The Gulf countries have barked up the wrong tree. Neither domestic self-sufficiency nor self-sufficiency by proxy (i.e. farmland abroad) are the main challenges, rather the management of value chains, food diplomacy to ensure the functionality of multilateral frameworks, tackling malnutrition and its consequences (e.g. obesity) and ensuring food accessibility for vulnerable segments of the population – such as migrant labour and people with insecure residency statuses.

## Self-sufficiency and land investments as failed strategies

The MENA region lost its ability to grow its required amounts of food from domestic renewable water resources in the 1970s already and has increasingly come to rely on food trade (Allan 2003). There is unease with this growing dependency, which can be explained by a number of negative historic experiences: the difficult food supply situation in World War II, the politicisation of food trade by the United States in the 1960s and 1970s when it used subsidised Public Law 480 food exports for political leverage, the same country’s threat of a food embargo in retaliation for the Arab oil boycott and, more recently, sanction regimes like that of the United Nations in Iraq from 1990 to 2003 that wreaked havoc on food security in the latter country.

There are also intellectual arguments for a degree of self-sufficiency that run counter to the trade-based approach to food security that is advocated by economic liberalism and international organisations such as the World Bank. Countries that have limited purchasing power, rely on volatile export earnings, depend on a main staple food that is controlled by only a few suppliers (e.g. rice), or are at risk of sanctions, war and/or geopolitical supply disruptions might benefit from reducing their exposure to volatile international markets. Finally, heavy reliance on imported calories is simply not an option for vast countries such as China and India because of their sheer size in comparison to exportable surpluses elsewhere (Clapp 2017).

Gulf self-sufficiency aspirations are understandable, but have clashed with the realities of resource constraints and population growth. Agricultural subsidies made Saudi Arabia the world’s sixth-largest exporter of wheat in the 1990s, but it had to phase out production between 2008 and 2016 for lack of water. Domestic agricultural aspirations nowadays confine themselves to the water-efficient cultivation of fruit and vegetables in greenhouses and vertical farms as well as livestock-raising with imported fodder – not the grandiose self-sufficiency visions of yesteryears.

Farmland investment abroad, the self-sufficiency by proxy strategy, has failed as well. Announcements were grandiose and quickly became part of a media narrative about the Gulf countries buying up Africa and other developing countries. But implementation lagged behind, mimicking the failure of a similar plan in the 1970s to develop Sudan into an Arab bread basket (Woertz 2013). Lack of infrastructure, a challenging local business environment, missing expertise on the part of the Gulf countries, resistance by disenfranchised holders of customary land rights and reduced fiscal space to invest as a result of falling oil prices all took their toll. The farmland-investment wave has not made its mark in the food-trade statistics over a decade after its launch: The Gulf countries continue to receive the vast majority of their imported food from established agro exporters, among them a growing number from tropical regions such as Brazil. Major target countries of land investments in the developing world such as Sudan hardly figure meanwhile (International Trade Center 2020; Woertz and Keulertz 2015).

## Management of value chains

If the Gulf countries did actually put money on the table it was mostly in developed agro markets and investments were in food-trade value chains rather than further upstream in farmland. Saudi state-owned SALIC, for example, teamed up with international grain-trader Bunge to purchase a majority stake in the privatised Canadian Wheat Board. The Gulf countries themselves are home to modern value chains in food processing and distribution. Gulf capitalists play an influential role in such modern value chains in other MENA countries, too, operating via large conglomerates such as Kuwait-based Americana and Saudi Arabia-based Savola (Hanieh 2018).

The impact of COVID-19 will most likely be most severe in traditional and transitional food value chains, such as household microenterprises on the one hand and small and medium-sized enterprises and wet markets on the other (Reardon et al. 2020). Informal and labour-intensive, they bring together large numbers of workers in crowded spaces – at considerable risk of contagion and with ensuing shutdowns and labour shortages. The capital-intensive farming of staple crops will be less affected in comparison. The labour-intensive cultivation of fruit and vegetables will likely fare better as well, as it is subject to less labour density than the traditional and transitional downstream value chains. This all means that the Gulf countries find themselves in a relatively privileged position: their global supplies of agricultural products are unlikely to dry up in the foreseeable future, while their modern food value chains that are dominated by supermarkets and capital-intensive processing plants are less vulnerable to COVID-19-related disruptions.

Much of their reaction has already focussed on the management of value chains and their international linkages. They have increased storage to bridge supply shortfalls, diversified providers, streamlined sanitation measures and eased import flows by changing the requirement for all goods to be labelled in Arabic. Internationally, stocks of staple foods are higher and markets better supplied than during the global food crisis of 2007/08. Some food exporters such as Ukraine and Vietnam have announced quotas, but price spikes and outright export restrictions have not occurred so far. Political awareness about the detrimental effects of such restrictions has grown meanwhile.

Multilateral initiatives such as the Agricultural Markets Information System (AMIS) have sought to increase market transparency and dampen food-price volatility. Such initiatives could also help to limit national rushes to beef-up physical storage, which might create the very problem that they wish to address: illiquid food markets (von Braun et al. 2009). The Gulf countries may consider raising their concerns as well in multilateral bodies such as the World Trade Organization and the G20, not unlike the group of the Net Food-Importing Developing Countries (NFIDC) that formed an interest group within the General Agreement on Tariffs and Trade negotiations during the Uruguay round to lobby for affordable food imports (Narlikar 2003).

## Food accessibility and malnutrition

In contrast to the global food crisis, a renewed wave of announced land investments is not likely. The strategy has not brought benefits and plummeting oil prices mean that there is less capital available for adventurous investment deals. Securing food focusses now on the management of value chains, where the Gulf countries are in a relatively privileged position as a result of their modern food systems. As before, food availability is not the primary issue – having proven to be manageable.

However, COVID-19 has turned the spotlight onto issues of food accessibility and malnutrition. The Gulf countries have one of the largest per capita shares worldwide of people suffering from diabetes and obesity, two groups particularly at risk of succumbing to COVID-19. Authorities have been reluctant to address underlying issues more forcefully, such as sedentary lifestyles and diets rich in sugar, fat and carbohydrates (Alnohair 2014). The obesity epidemic, which is particularly prevalent among females, highlights that food security is rarely an issue of lacking calories, rather of insufficient micronutrients. This also shows in other phenomena related to nutrition insecurity. The percentage of children in the Gulf with stunted growth is smaller than elsewhere in the MENA region, but still in the higher single digits in Saudi Arabia, Bahrain and Oman (Breisinger et al. 2012).

Food accessibility can also be compromised for vulnerable segments of the population, namely migrant workers and those with insecure residency statuses. Migrants make up the vast majority of the population in the UAE (88%), Qatar (76%) and Kuwait (74%). In Saudi Arabia and Oman, they form substantial minorities – being 32% and 41% of the population, respectively. In Bahrain, their population share is 51% (World Bank 2020). Additionally, there are long-term residents with unclear residency status – the so-called *bidoon* (“without”). In Kuwait and the UAE, they often hail from local Bedouin populations that were not awarded citizenship status at the time of independence. In Saudi Arabia, large shantytowns exist around Mecca, Medina and Jeddah that are home to descendants of migrants from Africa and Southeast Asia who came to the country decades ago and overstayed their visas. Like migrant workers, they form a permanent underclass with limited social and legal rights.

COVID-19 is not the great leveller that will affect everybody equally. The poor are more at risk than the affluent. They can ill afford to shelter in place, their blue-collar occupations cannot be conducted from the safety of the home and they do not have meaningful savings to bridge longer periods of time without employment and income. Their cramped living quarters constitute an increased risk of contagion and they have less access to healthcare if they contract the disease.

COVID-19 has brought long-standing grievances in the Gulf labour sector into sharp relief, such as non- or late payment of wages, limited rights under the *kafala* sponsorship system and poor working conditions (Kamrava and Babar 2012; Rajan 2019). Migrant workers suffer most from the economic havoc that COVID-19 is wreaking. Current circumstances undermine their ability to purchase nutritious food in adequate quantities. National labour still works predominantly in the public sector, where it enjoys a degree of protection or receives relief aid from the government that is only available for nationals. In the private sector nationals also enjoy more protection. Saudi Arabia, for example, has decided to pay 60% of the private sector wages of Saudi nationals for up to three months as part of its Unemployment Insurance scheme (SANED). In comparison, the economic impact of COVID-19 is far more severe for migrant workers. They suffer most from restrictive measures like quarantines and their access to relief aid and healthcare is limited.

The recent decision by the Saudi government to open up the healthcare system to everybody for COVID-19 treatment regardless of their legal residency status is an implicit acknowledgement that such lack of access can be a public health risk. Maintaining food security under COVID-19 will require safety nets that go beyond random charity transfers. The necessary formalisation that goes with that will likely raise concerns. The issue of migration was a sensitive one even before COVID-19 and tied to debates on demographic imbalances and political stability. Already there are calls in Kuwait, Saudi Arabia and Oman to take the COVID-19 crisis as an opportunity to permanently reduce the share of migrants in the overall population. The UAE and Qatar have relaxed their flight bans, allowing outgoing flights to repatriate migrant workers. Many migrant workers are suffering with regard to food accessibility, as they find themselves caught between a rock and a hard place. Either they stay in the host state with no or reduced pay or they return prematurely to their home countries, with their limited savings gone and struggling to repay the fees of the agencies that facilitated their temporary labour migration in the first place.

## Conclusion

The food systems of the Arab Gulf countries have so far performed well during the COVID-19 crisis in terms of ensuring food availability. Their modern value chains are more resilient than the traditional and transitional ones that dominate in developing countries. The improvement of such value chains deserves the attention of policymakers and researchers alike. Hydroponics, aquaponics, vertical farming and other modern production technologies might allow the Gulf countries to meet some of their requirements regarding fruit, vegetables and fish without depleting groundwater resources at unsustainable rates. However, the vast majority of food supplies will continue to come from global markets. Multilateral frameworks such as the WTO and G20 could help in making international food trade more dependable, yet the Gulf countries have not paid enough attention to corresponding food diplomacy at the respective bodies.

The COVID-19 crisis has also highlighted that food security is nutrition security above all else. It could provide an opportunity for the Gulf countries to tackle food-related epidemics such as obesity and diabetes more forcefully. Policy interventions could range from taxation on and declaration requirements for carbohydrates and sugar-rich foods to school meals and awareness campaigns. Food accessibility for migrant workers and other vulnerable segments of the population is another priority area of concern. It would require improvement of existing labour regulations, assistance programmes and safety nets. As such, it points to the fraught debates around long-standing grievances in the labour sector that intersect with concerns about demographic imbalances and workforce nationalisation – political concerns that dim prospects of reform significantly.

## Data Availability

Not applicable
